# COQ8B-Related Steroid-Resistant Nephrotic Syndrome in Saudi Arabia: A Case Report

**DOI:** 10.7759/cureus.31922

**Published:** 2022-11-26

**Authors:** Nasser H Alvi, Bakur A Turkstani, Ahmad S Ashi, Abdullah M Alzahrani, Abdulaziz M Tawffeq

**Affiliations:** 1 Family Medicine, Ministry of National Guard-Health Affairs, Jeddah, SAU; 2 College of Medicine, King Saud Bin Abdulaziz University for Health Sciences, Jeddah, SAU

**Keywords:** coenzyme q10, genetic mutation, nephropathy, steroid resistant nephrotic syndrome, coq8b, coenzyme q8b

## Abstract

The CoQ10 enzyme has several vital roles in the human body. CoQ10 deficiency can lead to many clinical manifestations including the steroid-resistant nephrotic syndrome. At least 16 genes work together to facilitate the correct synthesis of CoQ10, one of which is *CoQ8B*. We report the case of a 14-year-old male with a rare homozygous variant, who presented with late severe nephrotic syndrome and bilateral small dysplastic kidneys. This report will also describe the comprehensive and systemic workup that is needed in these patients. We conclude that physicians need to consider renal causes in their workup of any unexplained oedema in children and that in such cases, screening for rarer genetic causes should be considered in a country such as Saudi Arabia, given the relatively high rates of consanguinity here.

## Introduction

Quinones are a class of compounds that consist of a mono or polycyclic aromatic system, to which a dione is conjugated. Coenzyme Q is a lipophilic molecule existing in humans whose principal function is to transport electrons in the mitochondrial respiratory chain to create adenosine triphosphate (ATP). It has many lesser-known yet crucial functions as an antioxidant and also participates in cell signaling [1]. Coenzyme Q exists mostly in humans in a polyisoprenoid form of 10 units (CoQ10). However, its correct biosynthesis depends on several genes, including the *CoQ8B* (*ADCK4*) gene. One of the many possible clinical presentations of CoQ10 deficiency is renal involvement, manifesting as a steroid-resistant nephrotic syndrome (SNRS) [2]. In this report, we present the diagnosis and treatment of a child who presented with SRNS due to a rare pathological homozygous variant of the *COQ8B* gene, which, to our knowledge, has only been reported once before.

## Case presentation

**Figure 1:**
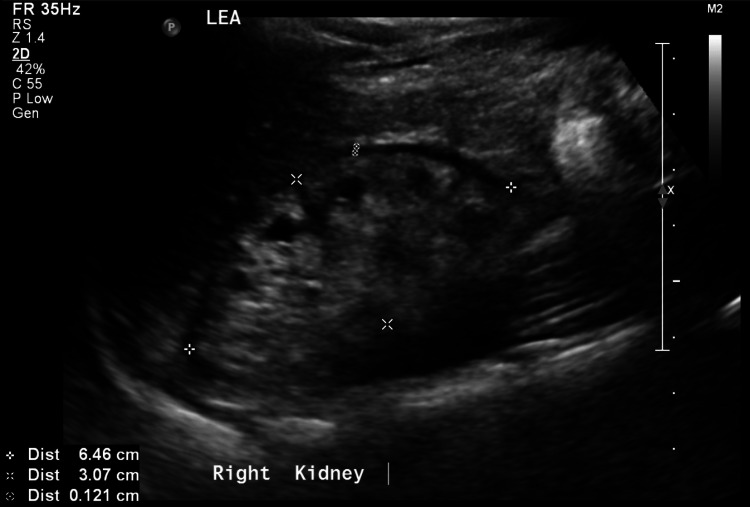
The right kidney showing a size of 6.46cm by 3.07cm

**Figure 2:**
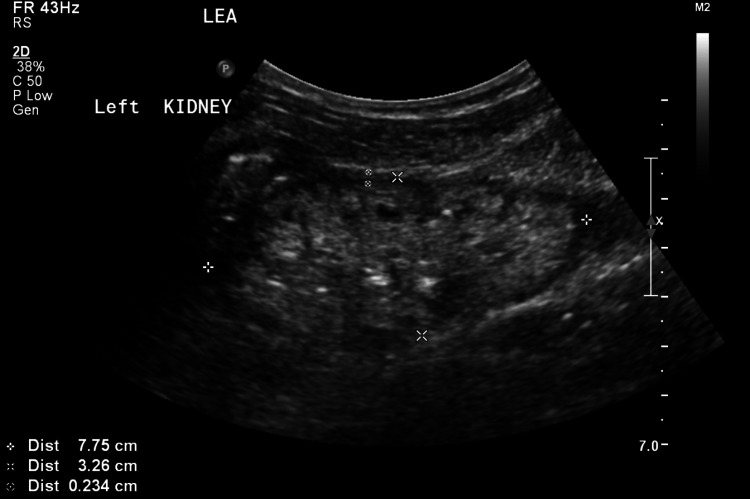
The left kidney showing a size of 7.75cm by 3.26cm

A 14-year-old boy was brought to a family medicine clinic in November 2020 complaining of vomiting and diarrhoea for one week. He was noticed to have facial and periorbital swelling of uncertain duration. During the initial triage, it was found that his blood pressure was 179/116 mmHg. The paediatrics team was consulted after which he was commenced on hydralazine alternating with nifedipine for blood pressure control. He was admitted to the general paediatrics ward for monitoring and further workup. A urinalysis was performed, which showed proteinuria 300 mg/dL, moderate hematuria, and a pH of 5.0. His creatinine level was 898 micromol (MMO)/L, potassium level was 5.2 mmol/L, and albumin level was 30 g/L. A kidney ultrasound was performed, which showed that both of his kidneys were in the fifth percentile in size for his age (Figures 1, 2). A diagnosis of chronic kidney disease secondary to bilateral small dysplastic kidneys was made.

During admission, the patient was given multiple medications to control his blood pressure including labetalol (60mg every 8 hours), amlodipine (10mg once daily), lisinopril (10mg once daily), furosemide (40 mg iv every 24 hours) and nifedipine alternating with hydralazine only if his blood pressure was 130/80 mmHg or higher. Despite this, his blood pressure remained uncontrolled and he had persistent proteinuria. A decision to start hemodialysis was made and the patient underwent hemodialysis three days a week for eight months. 

Given the potential for multiorgan involvement in such a disease, numerous examinations and imaging procedures were done to determine comorbidities and monitor for complications. From a cardiology standpoint, an electrocardiogram showed normal sinus rhythm. An echocardiogram was also performed, demonstrating mild concentric left ventricular hypertrophy, and mild mitral regurgitation, findings consistent with long-standing systemic hypertension. The ECHO excluded the possibility of heart failure. An ophthalmologic examination demonstrated bilateral optic nerve swelling. A brain MRI was done to exclude causes such as space-occupying lesions and manifested no evidence of any abnormality. Involvement of the lungs, gastrointestinal tract, genitourinary tract, hematological system, and endocrine system was excluded during subsequent follow-up visits in the outpatient clinic. Measurement of C3 and C4 complement showed a result of 1.31 g/L and 0.35 g/L respectively, thereby excluding the possibility of lupus nephritis. Other than optic nerve swelling, this patient did not show other extrarenal manifestations. For example, he did not show signs of encephalopathy or mental retardation, as his development was going through a normal rate and his clinical picture did not suggest otherwise.

After consulting the pediatric genetics team, four months after his admission, samples from the patient and both of his parents were taken for whole exome sequencing (WES) analysis. The patient’s parents were noted to be consanguineous. WES showed that the patient had a homozygous variant c.532C>T p.(Arg178Trp) in *COQ8B* (OMIM:615567), which leads to amino acid exchange. Sixteen out of 22 bioinformatic in silico programs predict a pathogenic effect for this variant. Parallel analysis of parental WES data revealed that both parents were heterozygous carriers of the detected variant in *COQ8B* (Table 1), thus confirming the homozygosity of the detected variant in the index.

**Table 1:** Gene variant with significant information COQ8B: Coenzyme Q8B; MIM: Mendelian Inheritance in Man; AR: Autosomal Recessive; chr: Chromosome; MAF: Mutation Annotation Format; gnomAD: Genome Aggregation Database; Arg: Arginine; Trp: Tryptophan; C: Cytosine; T: Thymine; P: protein

Classification	MAF gnomAD	Zygosity			Variant	Phenotype MIM number	Gene (isoform)
Pathogenic	0.0042	Father	Mother	Index	c.532C>T p.(Arg178Trp) chr19:41211045	615573 (AR)	COQ8B (NM_024876.4)
		Heterogeneous	Heterogeneous	Homogeneous			

Pathogenic variants in *COQ8B* cause autosomal recessive type 9 nephrotic syndrome (NPHS9; OMIM: 615573) characterized by significant proteinuria resulting in hypoalbuminemia and oedema. The disorder is steroid treatment-resistant and usually progresses to end-stage renal disease requiring renal transplantation.

Considering the homozygous pathogenic variant in *COQ8B* and the supportive phenotype of the patient a genetic diagnosis of SRNS secondary to *COQ8B* mutation was made four months after his first admissions.

As a result, the patient was started on COQ10 supplementations (120mg once daily). The dose of COQ10 supplementation was modified numerous times in response to the patient's clinical manifestations, blood pressure, and lab results during follow-ups at the clinic until it reached 600mg twice a day. A kidney biopsy was not done because a secondary cause of SRNS was identified; being a mutation in *COQ8B*.

The patient and family members were first counseled on the need for kidney transplantation during the first admission immediately after starting hemodialysis. There then followed a period of waiting until the appropriate donor was found. In August 2021, the patient underwent kidney transplantation, which was uneventful.

He remains on lisinopril and labetalol for hypertension control and also takes COQ10 supplementations. His last three blood pressure readings in the clinic have been at normal range and he has had no elevated creatinine levels. He is still under follow-up at the clinic for blood pressure measurements and monitoring for further complications.

The patient's improved clinical picture can be a result of the multifactorial management approach that was undertaken. He was put through renal replacement therapies; such as hemodialysis and kidney transplantation. Moreover, COQ10 supplementation was given to control COQ10 deficiency after the results of WES identified the specific etiology of the disease, which was determined to be secondary to *COQ8B* mutation.

## Discussion

COQ10 is synthesized on the mitochondrial membrane by a complex process that includes different enzymes, encoded in 15 variable genes [1], including *COQ8B/ADCK4* gene, which encodes a putative kinase that has a regulatory role in the synthesis of COQ10. A mutation in the *COQ8B* gene can therefore strongly reduce the levels and functions of COQ10 in affected individuals [2].*COQ8B/ADCK4* is located in chromosome 19q13.2 and has 15 axons that spread across 12 kb of human DNA [3]. *ACDK4* was reported to be located in cultured human podocytes [2].

COQ10 plays an important role in many cellular pathways, including the transportation of electrons in the mitochondrial membrane during aerobic cell respiration. Moreover, it is present in all cells as an antioxidant [4]. COQ10 deficiency secondary to *COQ8B* mutation can manifest clinically as SRNS leading to end-stage renal disease. However, primary COQ10 deficiency can manifest as multi-organ dysfunction. The spectrum of the clinical presentation includes encephalopathy, intellectual disability, peripheral neuropathy, SRNS, hypertrophic cardiomyopathy, retinopathy, sensorineural hearing loss, and muscle weakness. Moreover, it can lead to multiple complications such as end-stage renal disease and heart failure if not managed early. Patients with such a condition often need to undergo renal replacement therapies such as hemodialysis and renal transplantation. A patient that has refractory proteinuria should therefore have all the investigations and imaging procedures needed to rule out secondary diseases [5].

COQ8 B-related SRNS can be classified as a mitochondrial disease, a broad term covering several different diseases that are caused by mutations in the genes of enzymes responsible for essential mitochondrial processes. These diseases are rare and complex in nature. It is often necessary to investigate extensively when trying to diagnose and treat a patient from this category [6]. It has been estimated that the incidence of primary COQ10 deficiency is less than 1:100,000 [5].

Patients with COQ10 deficiency secondary to a mutation in *COQ8B* need to be started on COQ10 supplementation in order to compensate for the deficiency caused by *COQ8B* variant. Many patients will achieve improvement in their symptoms and quality of life. This has been demonstrated by a study by Korkmaz et al., which reported that two out of 26 patients with early-stage COQ8B nephropathy showed a reduction of proteinuria by 50% and 80%, respectively, within 6 weeks of COQ10 supplementation therapy that was given early after the diagnosis [7]. The study also highlighted the fact that a delay in the administration of COQ10 supplementation can delay recovery.. Moreover, Ashraf et al. reported that one out of 15 patients had partial remission after starting COQ10 supplementation following the diagnosis of SRNS secondary to a homozygous *ADCK4* variant [2]. In addition, the administration of COQ10 supplementation in the early course of the disease can accelerate recovery by decreasing proteinuria and normalizing kidney function [8]. These studies serve to emphasize the importance of COQ10 supplementation in treating such cases and that early detection and treatment are likely to be advantageous for patients' health and recovery. 

In our case, the patient was given COQ10 supplementation at a relatively late stage of the disease, four months after starting hemodialysis for three days a week. He was first given 120 mg once daily dose of COQ10 supplementation. The dose was modified and increased several times depending on the presence of proteinuria and blood pressure readings until it reached 600 mg twice a day. After six months, he had a successful renal transplantation. As a result of this multifactorial management approach, the patient showed a good response to therapy. His last three blood pressure measurements were within normal range and he demonstrated no elevated creatinine levels. However, more prompt investigations and proper management might have prevented the patient from going through renal replacement therapies.

## Conclusions

We report a case of SRNS secondary to *COQ8B* mutation that showed a good response to renal replacement therapies, such as hemodialysis and renal transplantation. COQ10 supplementation was given after the exact etiology of the disease was recognized. The patient presented with vague symptoms to primary care but his case was picked up by careful examination and attention to vital signs. His was a rare disease that may present at various stages of a patient's life with a variety of symptoms and lead to multiple complications. It is therefore imperative for physicians to be alert to the possibility of renal disease when children or young adults present with any abnormal swelling, blood pressure reading, or urinalysis. Genetic causes need to be actively looked into in these cases occurring in a country such as Saudi Arabia where consanguineous marriages remain relatively common.
